# Supplementary material for: Prevalence and determinants of food insecurity among pregnant women in Nigeria: A multilevel mixed effects analysis
Source: PLOS Glob Public Health. 2023 Oct 18;3(10):e0002363. doi: 10.1371/journal.pgph.0002363 (PMC10584166; doi:10.1371/journal.pgph.0002363)
Supplement: Attachment — Submitted filename: FIS_Nig_Preg_Authors response_Plos GH.docx [file pgph.0002363.s001.docx]

Review Comments to the Author

Reviewer #1: Overall, this study investigating the prevalence of household food insecurity and its association with individual- and contextual-level factors among pregnant women in Nigeria is both timely and important, given the potential negative impacts of food insecurity on maternal and child health. The methodology appears appropriate, utilizing a cross-sectional design based on the 2021 Nigerian Multiple Indicator Cluster Survey (MICS6) data and applying weighted multilevel multinomial logistic regression models to analyze the associations.

**Authors Response**

Thank you very much

**Reviewer #2:**

This manuscript from secondary data analysis of publicly available data majorly adhere to analysis and reporting of national level representative complex survey data.
The following clarifications/suggestions are recommended:

1. What all the factors being considered while estimating weighted %. (i.e Out of the factors such as stratification, clustering and Non Response rate, weightage mentioned in this manuscript refers to what?)

**Author’s response**

In order to account for the complex survey design (weighting, clustering, and stratification), we used the national women's sample survey weights for reporting survey results.

2. Does the survey design include more than one pregnant women in a family? In that case, the nested multi-level modelling has to address the household level factors as well before adjusting for community level factors?

**Author’s response**

Within each household, if there was more than one eligible respondent, only one respondent was selected randomly for interview (See <https://www.unicef.org/nigeria/media/6316/file/2021%20MICS%20full%20report%20.pdf>). Therefore, we merged individual-level and household-level factors and do not consider it absolutely necessary to address household-level factors separately in the analysis since we also included household factors such as household wealth index, number of household members and number of children under 5 years in household.

3. As the community level poverty index is in turn derived from individual household from the same community cluster, there is a possibility of multi collinearity? Did authors check for any collinearity while developing the model?

**Author’s response**

Yes, we checked for multicollinearity was checked prior to building our multilevel models. There was no multicollinearity identified as variance inflation factor (VIF) values were less than the threshold value of 10. We have made this explicit in the revised manuscript.

4. As given in the manuscript, dependent variable has ordinal categories. In this case the reason for using multinomial regression could be explained. While developing multinomial regression model, how did the assumptions of independent irrelevant alternatives were followed?

**Author’s response**

Thank you for your insightful comments. We appreciate your attention to detail regarding the choice of using multinomial regression for our dependent variable with ordinal categories.

In our study, we opted for the multinomial regression model due to its suitability for analyzing outcomes with more than two categories, especially when the categories have a meaningful order. This allows us to account for the inherent ordinal nature of the dependent variable while considering various predictors simultaneously.

To ensure that parameter estimates are unbiased and consistent, we tested for the independence of irrelevant assumptions (IIA), using the Small-Hsiao test, which evaluates whether belonging to one food insecurity category does not affect the other available categories We found no evidence to suggest violation of the IIA in the best fitting model. We have included this in the revised manuscript as well.

It is noteworthy that we are also aware of the argument put forward by Paul Allison on the caution not to rely on the IIA assumption for several reasons (See: <https://statisticalhorizons.com/iia/>) .

5. What is the extent of missing data on food insecurity measures among eligible pregnant women?

**Author’s response**

We excluded observations with missing or "Don't know" responses on food insecurity experience from the analysis (*n* = 46), resulting in a final analytical unweighted sample of 3519 pregnant women nested in 1318 primary sampling units. We have clarified this in the revised manuscript.

6. To understand how the geographical location (region) has affected food insecurity, it is better to describe the contextual factors (especially those having high food insecurity) associated with these regions.

**Author’s response**

We have described the contextual factors that could explain the protective effect of residing in the North Western region against food insecurity among pregnant women in Nigeria.

7. Similar to health insurance coverage, access to other social supportive measures (food security programs) and coverage for ANC services would also have influenced the food security level (The current model does not include these factors).

**Author’s response**

While we do agree that access to other social supportive measures (food security programs) and coverage for ANC services could also influence food security level among pregnant women, the Multiple Indicator Cluster Survey (MICS) used for this study does not capture these variables.

8. As reported in previous literatures gender dimensions and cultural norms play a major role in food security measures especially among women. This survey data directly may/may not have these data. However, speculations under these dimensions in discussion could enhance the understanding.

**Author’s response**

While we do agree that gender dimensions and cultural norms could also influence food security level among pregnant women, the Multiple Indicator Cluster Survey (MICS) used for this study does not capture these variables. We have however cautiously discussed this to some extent in the revised manuscript to avoid over-speculation.

**Reviewer #3:**

Abstract
1. Authors need to emphasise on the problem statement at the introduction section

**Author’s response**:

We have emphasized this in the revised manuscript.

2. Include the sample size

**Author’s response**:

We have included this in the revised manuscript. Our study was based on a secondary analysis using an unweighted convenience sample of 3519 pregnant women in Nigeria.

**Introduction**
**Reviewer’s comments**

Authors should be specific on the interventions and programs and list some of the efforts and developmental achievement.

**Author’s response**

We have included programs like the Supplemental Nutrition Assistance Program (SNAP) and the Special Supplemental Nutrition Program for Women, Infants, and Children (WIC) in the revised manuscript. In addition, we included the food security package (FSP) program and the food-for-work (FFW) program implemented in Ethiopia.

**Reviewer’s comments**

The gap for the study is not clear. They should strengthen it.

**Author’s response**

The gaps in knowledge have been strengthened in the revised manuscript.

**Reviewer’s comments**

There should be a theory guiding the study

**Author’s response**
We have included a theoretical framework for the study in the revised manuscript. In our study, we used Bronfenbrenner’s social-ecological model (SEM) as the framework for guiding our understanding of the complex interplay of factors associated with food insecurity among pregnant women in Nigeria.

**Methodology**
Why is marital status categorised as “yes” and “no”? What does yes and no mean

**Author’s response**

Thank you for drawing our attention to this. We have corrected this and made more explicit the meaning of marital status to reflect married or cohabiting (yes vs. no).

They should include education of mother, women empowerment questions and some household and paternal characteristics

**Author’s response**

The selection of covariates for the models was determined *a priori* through a comprehensive literature review, considering their biological plausibility in the exposure-outcome relationship, and the availability of variables in the survey data. While we desired to included maternal education in our analysis, most of the observations were missing for this variable and therefore, rather than using multiple imputation, we decided to exclude this variable from the analysis in our not to bias our results. However, some household characteristics such as household wealth index, number of children in household and number of household members were already included in the analysis as explicitly shown in Table 2.

Results and discussion

They should link the theory to the discussions

**Author’s response**

We have linked the social-ecological model to our discussions in the revised manuscript.

The recommendation is not strong. They should strengthen it

**Author’s response**

We have strengthened our recommendations in the revised manuscript
